# Does the treatment of anxiety in children with Attention-Deficit/Hyperactivity Disorder (ADHD) using cognitive behavioral therapy improve child and family outcomes? Protocol for a randomized controlled trial

**DOI:** 10.1186/s12888-019-2276-3

**Published:** 2019-11-13

**Authors:** Emma Sciberras, Daryl Efron, Pooja Patel, Melissa Mulraney, Katherine J. Lee, Cathy Mihalopoulos, Lidia Engel, Ronald M. Rapee, Vicki Anderson, Jan M. Nicholson, Rachel Schembri, Harriet Hiscock

**Affiliations:** 1 School of Psychology, Deakin University, Geelong, Vic Australia; 2 Murdoch Children’s Research Institute, Parkville, Vic Australia; 3 University of Melbourne, Parkville, Vic Australia; 4 The Royal Children’s Hospital, Parkville, Vic Australia; 5 Centre for Emotional Health, Macquarie University, Sydney, NSW Australia; 6 Judith Lumley Centre, La Trobe University, Bundoora, Vic Australia

**Keywords:** ADHD, Anxiety, Child, Randomized controlled trial, Efficacy, Treatment

## Abstract

**Background:**

Up to 60% of children with Attention-Deficit/Hyperactivity Disorder (ADHD) meet diagnostic criteria for at least one anxiety disorder, including Social, Generalized and/or Separation Disorder. Anxiety in children with ADHD has been shown to be associated with poorer child and family functioning. Small pilot studies suggest that treating anxiety in children with ADHD using cognitive-behavioral therapy (CBT) has promising benefits. In a fully powered randomized controlled trial (RCT), we aim to investigate the efficacy of an existing CBT intervention adapted for children with ADHD and comorbid anxiety compared with usual care.

**Methods:**

This RCT is recruiting children aged 8–12 years (*N* = 228) from pediatrician practices in Victoria, Australia. Eligibility criteria include meeting full diagnostic criteria for ADHD and at least one anxiety disorder (Generalized, Separation or Social). Eligible children are randomized to receive a 10 session CBT intervention (Cool Kids) versus usual clinical care from their pediatrician. The intervention focuses on building child and parent skills and strategies to manage anxiety and associated impairments including cognitive restructuring and graded exposure. Minor adaptations have been made to the delivery of the intervention to meet the needs of children with ADHD including increased use of visual materials and breaks between activities. The primary outcome is change in the proportion of children meeting diagnostic criteria for an anxiety disorder at 5 months randomization. This will be assessed via diagnostic interview with the child’s parent (Anxiety Disorders Interview Schedule for Children V) conducted by a researcher blinded to intervention condition. Secondary outcomes include a range of child (e.g., anxiety symptoms, ADHD severity, behavior, quality of life, sleep, cognitive functioning, school attendance) and parent (e.g., mental health, parenting behaviors, work attendance) domains of functioning assessed at 5 and 12 months post-randomization. Outcomes will be analyzed using logistic and mixed effects regression.

**Discussion:**

The results from this study will provide evidence on whether treating comorbid anxiety in children with ADHD using a CBT approach leads to improvements in anxiety and/or broader functional outcomes.

**Trial registration:**

This trial was prospectively registered: Current Controlled Trials ISRCTN59518816 (10.1186/ISRCTN59518816). The trial was first registered 29/9/15 and last updated 15/1/19.

## Background

Attention-Deficit/Hyperactivity Disorder (ADHD) is a highly prevalent neurodevelopmental disorder affecting 5% of the childhood population [1]. ADHD causes considerable burden for children, their families and society more broadly [2]. Children with ADHD often experience comorbid anxiety disorders, which contributes to poorer functioning [3]. Effectively treating anxiety disorders in children with ADHD may improve functioning, including overall quality of life, for children with ADHD [4]. This article describes the protocol for the Calm Kids randomized controlled trial (RCT), which aims to evaluate the efficacy of treating comorbid anxiety disorders in children with ADHD using a manualized cognitive behavioral therapy (CBT) approach compared with usual care.

### Anxiety in children with ADHD

A number of studies have demonstrated that children with ADHD are at increased risk of anxiety disorders compared to their peers [3]. For example, in a large Australian study of children with ADHD (*N* = 389), 64% of children with ADHD aged 5 to 13 years met diagnostic criteria for at least one anxiety disorder, including Social (48%), Generalized (34%), and Separation (32%) Anxiety Disorders [5]. In children with ADHD, anxiety emerges early in development with 25% of 6–8 year old children with ADHD meeting criteria for an anxiety disorder (assessed via diagnostic interview) compared with 8% of children without ADHD [6].

It is clear that anxiety has an independent impact on functioning for children with ADHD. Anxiety in children with ADHD is associated with poorer attentional and executive functioning, [7, 8] social functioning, [9] and more strained family functioning (e.g., poorer parental mental health, less positive parenting behaviors) [10]. There are also established links between internalizing difficulties, including anxiety, and sleep problems in children with ADHD [11–13]. In a previous study, our group found that children with ADHD who met criteria for multiple anxiety disorders also had poorer quality of life (QoL) (effect size [ES]: − 0.8), behavior (ES: 0.4), and daily functioning (ES: 0.3) compared to children with ADHD alone [5]. Children with ADHD and multiple anxiety disorders also had higher levels of school absenteeism [5].

There appears to be a significant under-identification of anxiety in children with ADHD in clinical practice. In a large study examining pediatric consultations for ADHD (*n* = 1528), only 8% of children with ADHD were reported to have comorbid anxiety, [14] which is well below estimates of prevalence from previous studies [3]. When identified, anxiety in children with ADHD may be treated with anxiolytic medications [15]. However, psychological therapies may be preferable given the potential side effects of medications, as well as the risk of drug interactions with multiple psychotropic medications [16]. In addition, parents may be reluctant to commence pharmacological treatment for ADHD and associated comorbidities [17].

### CBT for anxiety in children with ADHD

In the general population, there is strong empirical support for a variety of skills-based strategies and packages (generally referred to as CBT) for the management of pediatric anxiety [18]. A meta-analysis found moderate effects from such interventions over waitlist conditions with anxiety remitting in 59% of children following treatment with CBT compared with 18% in waitlist controls [19]. A recent study of children aged 5 to 18 years (*N* = 842) with an anxiety disorder, examined whether comorbid ADHD (*n* = 94) influenced treatment response to CBT [20]. The study found that ADHD did not predict treatment response or rates of remission for anxiety disorders and furthermore there were small improvements in ADHD symptoms for the ADHD group following CBT [20].

Few studies have examined whether CBT is efficacious in treating anxiety in children with ADHD and comorbid anxiety, with most of them being uncontrolled and including small sample sizes. Jarrett and Ollendick [21] reported improvements in anxiety and ADHD symptom severity immediately following a 10-week CBT program for children aged 8–12 years with comorbid ADHD and anxiety (*n* = 8). Similarly, two other small studies (*n* = 7–10), found improvements in anxiety following a CBT program for children and adolescents with ADHD [22, 23]. Although Costin et al. [24] reported high levels of parent and child satisfaction following an 8-week CBT program for 7 boys with ADHD, oppositional defiant disorder and anxiety, there was no measurable improvement in anxiety, which is likely due to the high level of comorbidity in this sample.

To our knowledge only one randomized controlled trial (RCT) has examined the treatment of anxiety in children with a primary diagnosis of ADHD using CBT [4]. This small pilot study (*n* = 12, aged 8–12 years) from our research team aimed to test the acceptability and feasibility of treating anxiety in children with ADHD using CBT (a program known as “Cool Kids”) compared with usual care. The trial had good uptake (67%) and the majority of families attended all 10 intervention sessions, with no drop-outs. At 5-months post-randomization, 3/6 of intervention children were free of an anxiety diagnosis (assessed via diagnostic interview administered by researchers blinded to intervention status) compared with 0/6 of controls. Intervention children had marked improvements in child and family wellbeing by parent- and teacher-report including anxiety symptoms (effect size (ES) = − 0.6), inattention symptoms (ES = − 1.1), QoL (ES = 0.6), behavior (ES = − 0.5) and parent mental health (ES = − 1.0) [4].

### Aims and hypotheses

This RCT aims to test the efficacy of an existing CBT intervention adapted for children aged 8–12 years with ADHD and comorbid anxiety compared with usual care. Eligible children will be randomized to the intervention group (10 CBT sessions over 12 weeks) versus usual clinical care. We hypothesize that, compared with the usual care group, the intervention group will have the following outcomes at 5 and 12 months post-randomization:

### Primary outcome

1. Reduction in the proportion of participants meeting criteria for an anxiety disorder assessed via a researcher blinded diagnostic interview at 5 months post-randomization.

### Secondary outcomes

1. Fewer anxiety symptoms, measured continuously, as reported by parents, children and teacher reports at 5 and 12 months post-randomization, as well as a reduction in the number meeting criteria for an anxiety disorder at 12 months post-randomization.
2. Reduced ADHD symptom severity at 5 and 12 months post-randomization.
3. Improved child behavior, quality of life, sleep, cognitive functioning, and school attendance at 5 and 12 months post-randomization.
4. Improved parent mental health, parenting behaviors, and work attendance at 5 and 12 months post-randomization.

We will also assess the cost-effectiveness of this intervention compared with usual care.

## Methods

### Overall study design

**Fig. 1:**
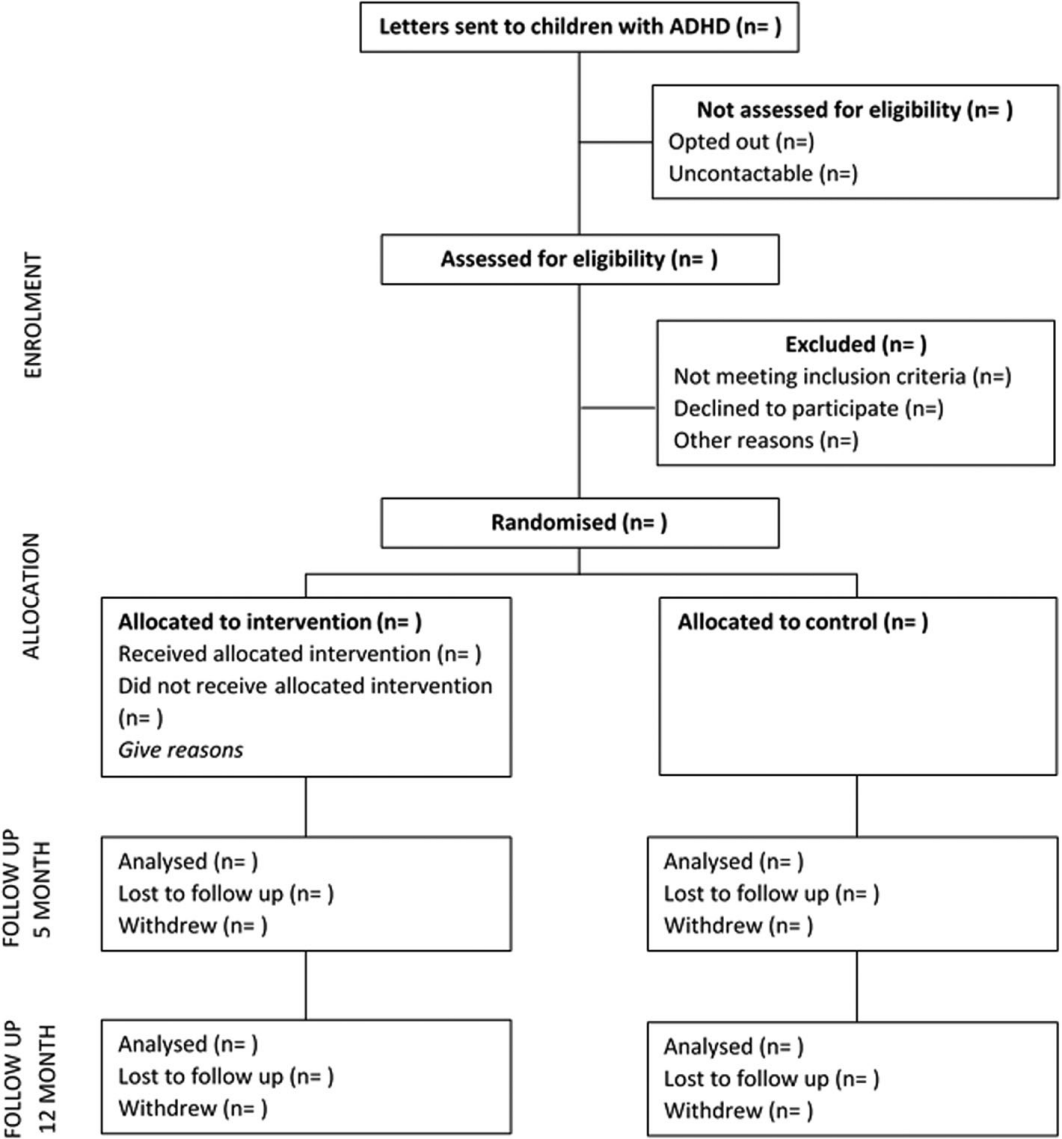
Participant Flow

This is a RCT of CBT versus usual clinical care conducted in the state of Victoria, Australia. The trial will be reported in accordance with CONSORT guidelines for RCTs. We have prepared this study protocol adhering to the SPIRIT checklist. This trial has been prospectively registered: Current Controlled Trials ISRCTN59518816. See Fig. 1 for the participant flowchart.

### Participants

Participants comprise families of children aged 8–12 years with diagnostically-confirmed ADHD (see inclusion criteria) who also meet diagnostic criteria for one or more of Separation, Generalized, and Social Anxiety Disorder.

### Screening children for anxiety and recruiting families

Participants are recruited from two sources: 1. an existing database of families who had expressed interest in hearing about ADHD research; and 2. paediatric outpatient clinics in public hospitals or private pediatrician rooms in metropolitan and regional Victoria, since ADHD is commonly managed by pediatricians in Australia [14]. Across both recruitment sources families of children aged 8–12 years with ADHD (irrespective of whether anxiety has been identified given that this may be under-recognized) are mailed study letters and information booklets. An ‘opt out’ approach is used for initial contact about the study, whereby parents opt out if they *do not* wish to be telephoned by the research team to learn more about the study. If parents do not opt out within 2 weeks, the research team then telephones families to explain the study, answer any questions, and ascertain if they are interested in participating. If they are interested in hearing more, the inclusion/exclusion criteria listed below are checked.

Eligible families complete a baseline assessment visit at The Royal Children’s Hospital, their pediatrician’s office, or family home depending on preference. At this visit the parent and child each complete an electronic survey, and the child completes a direct assessment of cognitive functioning. Parents complete a study consent form and are asked to also provide consents for: data collection from their child’s teacher; data linkage access their child’s National Assessment Program-Literacy and Numeracy (NAPLAN) scores (years 3, 5, & 7); and data linkage access to their child’s Medicare/Pharmaceutical Benefits Scheme data (Australian universal subsidized health care insurance scheme) for the economic evaluation. Medicare records include services that qualify for Medicare benefits (e.g., appointments with psychologists and pediatricians) and for which claims have been processed, whereas the Pharmaceutical Benefits Scheme collects information on the prescription medications that participants have filled at pharmacies. Parents are also asked whether they consent for their de-identified data to be used for use in future studies. If parental consent is provided, the school principal is notified and teachers are invited (via email) to complete baseline and follow-up surveys assessing the child’s behavior at school.

### Inclusion/exclusion criteria (all assessed via telephone)

To be included in the trial, the child needs to meet Diagnostic and Statistical Manual of Mental Disorders (DSM) criteria 5 [25] criteria for:

- ADHD: To assess this, parents are asked to rate their child’s behavior when not on stimulant medication using the 18-item ADHD Rating Scale IV - a validated scale measuring core ADHD symptoms [26]. As per DSM 5 criteria, [25] at least 6/9 inattention and/or 6/9 hyperactivity/impulsivity symptoms need to be endorsed by the parents, and the child needs to have symptom duration of at least 6 months, age of onset prior to 12 years, and evidence of impairment across settings; and
- Generalized, Social and/or Separation Anxiety Disorder: This is assessed using the Anxiety Disorders Interview Schedule for Children V [27]. In our previous studies, 97% of children with ADHD and a comorbid anxiety disorder had one or more of these anxiety disorders thus we are confident that our findings will be generalizable to most children with ADHD and anxiety [5, 6].

The following exclusion criteria are also applied: non-English speaking, major child illness or disability (e.g., intellectual disability), child currently receiving CBT for anxiety, or commenced an anxiolytic/anti-depressant medication within the preceding 6 weeks. Children will be excluded if they have high levels of Autism Spectrum Disorder symptoms as indicated by scores of 15 or higher on the Social Communication Questionnaire [28].

### Sample size

A Cochrane review reported on the efficacy of CBT for children and adolescents with anxiety disorders in the general population and found remission of anxiety diagnoses in 59% of CBT participants (*n* = 808) immediately post-intervention compared with 18% in controls (*n* = 542) [19]. Taking a conservative approach, we based our sample size on observing remission in 40% of the intervention group and 20% in the control group (odds ratio of 2.7) at 5 months post-randomization (approximates post-intervention point). In order to provide 80% power to detect this clinically meaningful difference, using a two sided test with alpha = 0.05 and accounting for 20% drop-out, we need to enrol 114 children in each arm (228 total children). This sample size will enable us to detect differences of 0.42 standard deviations (SDs) between the two groups with 80% power in the continuous secondary outcomes at 5 and 12 months (using a two-sided test with alpha = 0.05). Assuming that 10% of families opt out (based on our previous ADHD trials), 47% of those contacted are eligible, and 70% of those eligible consent to participate (based on our pilot study), we need to approach approximately 771 families in order to recruit the required sample of 228 children.

### Randomization

Upon receipt of the completed consent form and completion of baseline parent and child measures, families are randomized to either the CBT or the usual care (control) group. A randomization schedule was generated using block randomization with variable block sizes by an independent statistician in the Clinical Epidemiology and Biostatistics Unit at the Murdoch Children’s Research Institute. Randomization is stratified by child sex to ensure similar proportions of males and females across groups.

Research assistants telephone families randomized to the intervention group to schedule intervention sessions at The Royal Children’s Hospital, their paediatrician’s office, or another community-based setting (e.g., school) depending on the family’s preference. Control children continue to access usual care for ADHD and anxiety from their pediatrician, which may include some simple behavioral management but is unlikely to include formal CBT [29]. All families can access other services, which may include CBT, while enrolled in the study.

Due to the nature of the intervention, it is not possible to blind participants to their allocation. Our primary outcome is a researcher-blinded diagnostic interview and we also conduct direct assessments of cognitive functioning. The comparison to usual care is essential, as we aim to evaluate whether our intervention is more beneficial than care currently received in the community. Given the importance of demonstrating longer-term benefits associated with interventions, we have opted for a usual care comparison group over a waitlist control group. Based on our previous intervention trials and pilot RCT of this intervention we do not foresee any risks or adverse effects of the intervention. Participants will be free to cease the intervention and/or withdraw from the study at any time.

### Intervention content, training & delivery

#### Content

This project uses the “Cool Kids” CBT program to treat anxiety in children with ADHD. Cool Kids has been evaluated in more than 15 clinical trials including several internationally and is currently used to treat anxiety in over 20 countries. Up to 80% of children with anxiety who have completed the Cool Kids program were diagnosis free or had substantially improved immediately post-intervention, with benefits persisting for up to 6 years [30]. Cool Kids consists of 10 sessions over 12 weeks (8 × 1 h-long weekly sessions, followed by 2 × 1 h-long fortnightly sessions), and is delivered by trained psychologists to the child and their parent(s) together. The program builds child and parent skills and strategies to manage anxiety and associated impairments. There is a strong focus on home-based skills practice. Session content is detailed in Table 1.

**Table 1:** Session structure of the Cool Kids Program

*Session 1:* Psychoeducation, fears list/goals, worry scale, linking thoughts and feelings.	
*Session 2:* Detective/realistic thinking, rewards.	
*Session 3:* Detective/realistic thinking, design of first exposure hierarchy (stepladder).	
*Session 4:* Parenting an anxious child (parent only session).	
*Session 5*: Review of exposure progress, creative exposure, design of further stepladders.	
*Session 6*: Review of exposure progress, simplified realistic thinking, in-session exposure.	
*Session 7, 8 & 9*: Troubleshooting exposure, realistic thinking, parenting, in session exposure. Additional skills to facilitate progress: problem solving, relaxation, social skills, assertiveness, dealing with bullying, surfing emotions, testing predictions and expectations.	
*Session 10*: Review goals, maintenance of gains, setbacks, future plans.	

We have made minor adaptations to the delivery of the Cool Kids program based on our clinical expertise and experience in trials with patients with ADHD. Adaptations include: 1) use of an activity schedule and positive reinforcement to promote on-task behavior; 2) one-minute ‘brain breaks’ between activities; 3) shortening and repetition of instructions and key concepts; and 4) increased use of visual aids in sessions and at home to promote home-based skills practice.

#### Training and delivery

The intervention is delivered by study-employed psychologists (provisional, generalist or clinical). ES trains the psychologists in a 1 day workshop modelled on the training of the original Cool Kids Program. Training incorporates adult learning principles including provision of didactic information and discussion of case studies. A clinician manual was developed for the pilot, outlining the content adapted from Cool Kids for each session. As part of tzzhe intervention, parents and children each receive a Cool Kids client manual covering all program content. Sessions are conducted with individual families (children and their parents together) as we anticipated that this would better cater to the needs of children with ADHD and anxiety compared to a group format.

Treatment fidelity is assessed through: a) fortnightly supervision meetings with psychologists facilitated by ES and/or another senior clinical psychologist; b) a standardized consultation record kept by clinicians with a checklist of program activities to be completed for each session; and c) coding of a random 20% of video-recorded treatment sessions by external researchers.

### Follow-up

At 5 and 12 months post-randomization, the research team telephones parents to complete a researcher-blinded diagnostic interview [27] and to book a face-to-face assessment visit (same locations as described above). During these 60–90 min visits, parents and children complete surveys and a direct child assessment of cognitive functioning. The research team also e emails follow-up surveys to teachers at both time points.

### Measures

This project uses REDCap to collect survey and interview data from parents, children and teachers. Study measures are summarized in Table 2. The primary outcome is whether the child meets diagnostic criteria for one or more anxiety disorders (Separation, Social or Generalized Anxiety Disorder) measured by the Anxiety Disorders Interview Schedule for Children V at 5 months post-randomization [27]. This measure has been selected as it is rigorous, maps onto DSM criteria, and takes into account both anxiety symptom severity *and* impairment. This interview is administered by an assessor unaware of group allocation via telephone with a parent/carer parents [42]. The diagnostic interview is repeated at 12 months post-randomization.

**Table 2:** Study measures

Construct	Measure	Source^a^	T1^b^	T2^c^	T3^d^
Primary Outcome					
Anxiety diagnoses	*Anxiety Disorders Interview Schedule for Children V:* Separation, Social, & Generalised Anxiety Disorder [27].	P	x	x	x
Secondary Outcomes					
Anxiety symptoms and impact	*Spence Children’s Anxiety Scale:* Child (44-item) and parent (38-item) reported anxiety symptoms [31].	P, C	x	x	x
	*School Anxiety Scale:* 16-items assessing anxiety symptoms at school [32].	T	x	x	x
	*Child’s Anxiety Life Interference Scale:* Child (9-items) and parent (16-items) report [33].	P, C	x	x	x
ADHD	*ADHD Rating Scale IV:* 18-item validated scale measuring the core symptoms of ADHD [26].	P, T	x	x	x
Behavior	*Strengths and Difficulties Questionnaire:* 25-items assessing child behavior and peer problems [34].	P, T, C	x	x	x
Irritability	*Affective Reactivity Index*: A 6-item validated measure assessing child irritability [35].	P	x	x	x
QoL	*Child Health Utilities Index Nine Dimension*: Preference-based measure of health-related QoL [36, 37].	P, C	x	x	x
Sleep	Single item primary caregiver report of whether the child’s sleep is a problem (none, mild, moderate or severe) [38].	P	x	x	x
School attendance	Number of days late/missed [38].	P	x	x	x
Cognitive function	*NIH Toolbox Cognition Battery:* Computer- administered assessment of cognitive and executive function, attention, episodic/working memory, and processing speed [39].	D	x	x	x
Parent mental health	*Kessler 6 (K6):* A 6-item validated measure of adult psychological stress resulting in a total score range from no distress to severe distress [40].	P	x	x	x
Parenting	*Parenting behavior:* Scales assessing parenting consistency (6-items) and warmth (6-items) [41].	P	x	x	x
Work attendance	Number of days late/missed [38].	P	x	x	x

^a^ P = parent report, T = teacher report, C = child report (completed face-to-face), D = direct assessment; ^b^ T1 = baseline; ^c^ T2 = 5 months post-randomisation; ^d^ T3 = 12 months post-randomisation

We also assess a range of other outcomes via parent, child and teacher reports (see Table 2). In addition, the baseline parent survey collects family and child demographic and medical data (i.e., medication, diagnosed comorbidities). Service use and treatments accessed are also assessed at each time point. At all time points, we conduct a direct assessment of cognitive functioning (NIH Toolbox Cognition Battery) [39] administered by a researcher.

### Data management

We use rigorous data management procedures to ensure confidentiality is maintained. The majority of the data is collected online using REDCap and can only be accessed by the research team. Hardcopy data is securely stores in locked cabinets, only accessible by the research team. All hardcopy data is entered into REDCap and subjected to data checking to ensure accuracy of data entry.

### Data analysis

Primary analyses will follow the ‘intention to treat’ principle at the child level. To account for loss to follow-up, analysis of the primary outcome (presence of Separation, Social and/or Generalised Anxiety Disorder) and all secondary outcomes will be carried out using mixed effects regression. For each outcome, a single mixed effects model will be fitted incorporating baseline, 5 month and 12 month data, adjusted for gender (randomization stratification factor), using random effects to allow for the repeated measures within an individual. These models will fit separate random effects and error terms at each time point, and a separate fixed effect of treatment at each of the two post-randomization time points. This approach implicitly deals with missing data as it enables all participants with baseline data to be included in the analysis.

For the primary outcome and other binary outcomes, groups will be compared using mixed effects logistic regression, with results presented as odds ratios and their 95% confidence intervals [CI] at 5 and 12 months. For continuous and count data secondary outcomes, groups will be compared using linear or Poisson mixed effects regression, as appropriate, with results presented as mean differences (and 95% CIs) in outcomes at 5 and 12 months. In addition, we will repeat the analysis adjusted for baseline covariates as appropriate: for example, child sex (male, female), child age, medication use (yes/no), comorbidities (yes/no), therapist, and family socioeconomic status). For our primary outcome, whether the child meets criteria for an anxiety disorder (separation, social or generalised anxiety diagnosis) at 5 months, a sensitivity analysis will be conducted using multiple imputation to handle the missing data with analysis via logistic regression applied to the 5 month data only.

### Health economics analysis

We will determine the cost-effectiveness of the Calm Kids intervention compared with usual care ‘within-trial’ from a health care perspective and a broader societal perspective. The collection of data on the Child Health Utilities Index Nine Dimension (CHU-9D) [36, 37] will enable the calculation of quality-adjusted life years (QALYs) that will form a cost-utility analysis. Health state preference values for the CHU-9D will be based on the value set derived by Ratcliffe et al., reflecting Australian adolescents’ preferences [43, 44], which will be combined with the period of the trial to derive QALYs. The cost-utility analysis will be complemented with a cost-effectiveness analysis, using the primary outcome measure of whether the child meets criteria for Separation, Social and/or Generalised Anxiety Disorder at 5 months post-randomization. This approach has been found to be useful to decision-makers [45]. Key costs and their measure (s) include: i) intervention costs: project team financial records; ii) costs accrued outside the intervention: parent-reported resource use questionnaire (baseline, 5 months and 12 months), adapted to reflect services used by children with ADHD (including community-based services, hospitalizations, out-of-pocket costs and parental productivity costs); iii) accurate and detailed health service usage for medical, pathology, diagnostic and pharmaceutical services: linked Medicare/Pharmaceutical Benefits Scheme data. Resource use and unit costs will be combined to derive a total cost for each participant. For both groups, mean values of costs and outcomes will be reported, as well as mean differences between the groups. The analyses will adopt both a health sector costing perspective (all health related costs will be included) as well as a broader partial societal perspective (parental work productivity also included). Generalized linear models will be used to assess mean difference in costs and outcomes in the two groups, using the same approach to confounding as described above. An incremental cost-effectiveness ratio (ICER) will be calculated as the difference in average cost between the groups, divided by the difference in average QALYs. Uncertainty in the data will be dealt with using nonparametric bootstrapping from the distribution of the observed cost/QALY pairs (1000 simulated replications) to determine CIs and presented in a cost-effectiveness plane along with a cost-effectiveness acceptability curve. To explore the robustness of the results, sensitivity analyses will be carried out (e.g., complete case analysis and unit cost variation).

## Discussion

The results from this study will provide evidence on whether treating comorbid anxiety in children with ADHD using a CBT approach leads to improvements in anxiety and broader functional outcomes compared with standard care. If the intervention is found to be efficacious, the evidence from this trial will inform efforts to test the translation of this intervention approach into clinical practice. We will publish our results in peer reviewed journals, present at conferences in this field and disseminate findings through professional associations (e.g., psychology, pediatrician and psychiatry associations) and ADHD organizations around the world. Eligibility for authorship will be in accordance with the Australian Code for the Responsible Conduct of Research. We also hope that our work will inform adaptations to existing clinical guidelines on the assessment and management of anxiety when it co-occurs with ADHD.

## Supplementary information

**Additional file 1.** Parent/Guardian Information Statement and Consent Form.

## Data Availability

Data collection is ongoing. We are collecting consent from participants for sharing de-identified data for future research. At the conclusion of the trial, requests to access the data can be made by emailing the corresponding author.

## Abbreviations

ADHD: Attention-Deficit/Hyperactivity Disorder
CBT: Cognitive-behavioral therapy
CHU-9D: Child Health Utilities Index Nine Dimension
DSM: Diagnostic and Statistical Manual of Mental Disorders
ES: Effect size
ICER: Incremental cost-effectiveness ratio
K6: Kessler 6
MCRI: Murdoch Children’s Research Institute
NAPLAN: National Assessment Program-Literacy and Numeracy
NHMRC: National Health and Medical Research Council
QALY: Quality-adjusted life years
RCT: Randomized controlled trial
SD: Standard deviation
