# Supplementary material for: Does the treatment of anxiety in children with Attention-Deficit/Hyperactivity Disorder (ADHD) using cognitive behavioral therapy improve child and family outcomes? Protocol for a randomized controlled trial
Source: BMC Psychiatry. 2019 Nov 13;19:359. doi: 10.1186/s12888-019-2276-3 (PMC6854614; doi:10.1186/s12888-019-2276-3)
Supplement: Supplementary file 1 — Additional file 1. Parent/Guardian Information Statement and Consent Form. [file 12888_2019_2276_MOESM1_ESM.pdf]

Murdoch Childrens Research Institute
Healthier Kids. Healthier Future.

Calm Kids Study

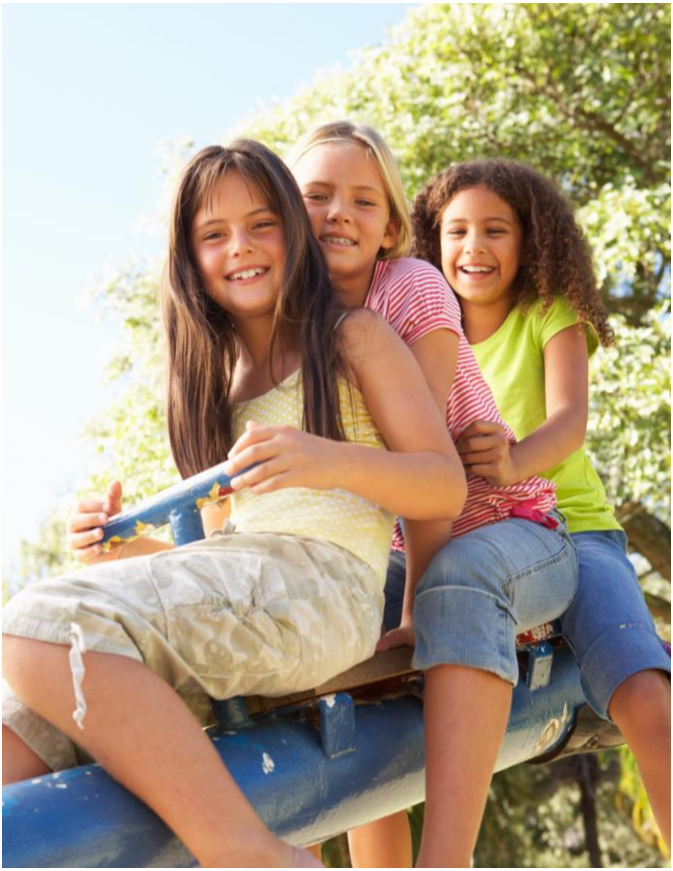

Parent/Guardian Information Statement and Consent Form

The Children's
Excellence in clinical care, research and education

Murdoch Childrens Research Institute
The Royal Children's Hospital
Flemington Road, Parkville VIC 3052 Australia
T 03 8341 6200 · F 03 9348 1391
ABN 21 006 566 972
www.mcri.edu.au

Chairman Leigh Clifford AO
Director Professor Kathryn North AM MD FRACP
Ambassador Sarah Murdoch

Dear Parents/Guardians,

Thank you for your interest in the Calm Kids Study! This is an important project which looks at whether managing anxiety in children with ADHD can improve child wellbeing.

You will receive a phone call from a member the Calm Kids team to talk about the project. They will ask you a few questions to see if the project is suitable for you and your child. If the project is suitable for your family, you will be invited to participate in **Calm Kids.**

Before you decide to take part, it's important to understand why we're running the study and what is involved. If you're invited to participate in the study, please take the time to read this booklet carefully, and discuss it with others if you wish.

At the end of this booklet there are two copies of the consent form. If you and your child are invited to participate in Calm Kids, and you decide to take part, we will ask you to sign and date both copies, and send one copy back to the Calm Kids project team.

Important things you need to know:

- It is your choice whether or not your child can take part in the research. You do not have to agree if you do not want to
- If you decide you do not want your child to take part, it will not affect the treatment and care your child gets at The Royal Children's Hospital

If anything isn't clear, or you would like more information please call us on (03) 9936 6306 or email us at calm.kids@mcri.edu.au. There will be more chances to ask any questions that you might have over the telephone, and on the assessment day before we begin the activities

Thank you for taking the time to consider taking part in the **Calm Kids** study**.** This information is also on our website https://www.mcri.edu.au/the-calm-kids-study.

1. What is the Calm Kids research study about?

This exciting research project is about anxiety in children with Attention Deficit Hyperactivity Disorder (ADHD). A large number of children with ADHD also experience anxiety (25-50%). We know that anxiety in children with ADHD makes daily life harder for children and their families.

The **Calm Kids** Study aims to see whether treating anxiety in children with ADHD improves child anxiety, as well as broader child and family functioning. To know whether the **Calm Kids** program helps, we need to compare children who receive the program with children who do not. To do this in a fair way we will randomly place children in either the 'Intervention Group' or the 'Usual Care Group'. This will be done by chance, similar to tossing a coin, so you will have an equal chance of being in either group.

The intervention we will be using to treat anxiety is the 'Cool Kids Program'. This is an intervention that has been very successful in the treatment of anxiety for children in the general community. It involves, over the course of 10 sessions, teaching children and parents what anxiety is, what causes anxiety, and what children and parents can do to lessen anxiety.

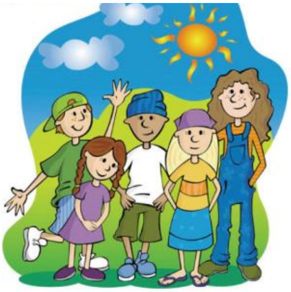

We hope that 228 children with ADHD and their families will participate in this project – we would love for you and your child to be involved!

2. Who is funding this research project?

The research is funded by the Besen Family Trust, the Myer Foundation and the National Health and Medical Research Council (NHMRC).

3. Why are we being asked to take part in Calm Kids?

We are inviting you to learn about this project because your child has ADHD and is aged between 8 and 12 years. We will ask you and your child to take part in the study if you indicate that your child is experiencing anxiety when we talk on the phone.

4. What does taking part in the Calm Kids study involve?

At the beginning, we would like you to complete a survey about your child's ADHD, and about your child and family's wellbeing. This will take about 20 minutes to complete. At this time we would also like to visit you and your child. This visit can be at home or you can visit us at the Royal Children's Hospital.

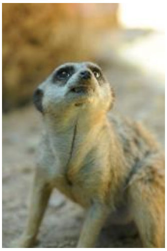

The visit will take about 60 minutes to do. The visit will include:

- **Completing a survey with your child about their own behaviour and feelings**
- **Activities that assess your child's thinking and language skills.**

Most children enjoy completing the assessment. We will provide you with a brief report from this assessment if requested.

Once we have received your survey and signed consent form, you will be randomly placed in one of two groups. What you will do next will depend on which group you are allocated to.

Usual Care Group

If you and your child are randomly placed into the usual care group, we will send you a letter to let you know which group you have been placed into. At this time we will also call you to ask you some questions about your child's emotional and behavioural functioning (20-25 minutes).

If you are assigned to the usual care group you will not receive any intervention. You can however, still receive assistance as you usually would from any professionals in the community (e.g., paediatrician or psychologist).

Treatment Program Group

If you and your child are randomly placed into the treatment program group, we will call you to arrange appointment times for the treatment program. During this call we will also ask you some questions about your child's emotional and behavioural functioning (20-25 minutes).
The treatment sessions can take place at The Royal Children's Hospital or at your local paediatrician's office. Should you choose to come to The Royal Children's Hospital for the sessions, your parking costs will be reimbursed.

What do parents and kids do during the sessions?

The sessions are done one-to-one – there will be no other families at the sessions. The treatment program is called the "Cool Kids Program". The program aims to help families and children to learn about anxiety and worries and to develop the skills to be able to manage child anxiety.
The program consists of:

- **8 one hour weekly sessions, followed by**
- **2 fortnightly one-hour sessions**

You can still receive assistance as you usually would from any professionals in the community (e.g., paediatrician or psychologist)

Usual Care and Treatment Groups – All children

We will contact you again in 5 months and 12 months time and ask you and child to:

- Complete surveys about your child's ADHD and repeat the questions about your child's anxiety. We will also ask you about child and family wellbeing.
- Repeat the face-to-face assessment of your child's thinking and language skills.

5. What do I need to consent to, and do I need to agree to everything?

If you are invited to participate in the study, we will ask you (the parent or guardian) to complete two copies of the consent form at the end of this booklet – one for you to keep and one for the **Calm Kids** team. You can do this before or at the beginning of your first assessment. There are six parts to the consent. You can agree to none, some or all consent options for your child:

a) **General consent:** First, we will ask for your overall consent for your child and yourself to take part in **Calm Kids**. This overall consent doesn't include the consent options below (b – f).

b) **Contacting your child's school teacher:** We would like your permission to ask your child's teacher to fill out a short survey at 3 times about your child's behaviour. This will involve first contacting the school Principal and gaining their approval to ask your child's teacher to participate in the research.

   In case your child's teacher or Principal is not aware that your child has ADHD, we will not refer to ADHD in the information we send to your child's teacher and Principal. Instead, we will say that this is a project about managing worries in children. You don't have to consent to this if you do not want to. Please tick the box on the consent form to say if we can contact your child's teacher or not.

c) **Recontact for future research:** In the future, new research projects may be suitable for you. We would like your permission to contact you about these projects. We will give you information about new projects then. It is up to you whether or not you agree to take part in a future project.

d) **Keeping your information for future research:** The **Calm Kids** study will collect information about you and your child. We would like your permission to keep this information indefinitely and share this information with future ethically approved research projects related to ADHD and other neurodevelopmental disorders. All information kept and shared will not identify you or your family. We will not contact you if we use this information in the future.

e) **Consent to link with NAPLAN data.** We would like your permission to let us link to your child's NAPLAN results, to collect additional information that will help us compare how children with ADHD and anxiety are performing, in comparison to the general population. Your NAPLAN data will be stored using your child's coded ID number, within a secure database. Please tick the box on the consent form to say if we can link to your child's NAPLAN results.

f) **Accessing your child's healthcare and medicines data from the Department of Human Services (DHS).** This will tell us if improving anxiety can reduce health care costs. If **Calm Kids** helps children, this helps funding bodies decide whether to provide it to other children in the future. We are asking permission to examine your child's Medicare Benefits Schedule (MBS) and Pharmaceutical Benefits Scheme (PBS) records for the period between now and our 12 month follow up. These records describe all the health services your child has received from doctors, other health practitioners and hospitals, and all the prescription medicines received during this period. If you agree to this we will get you to complete a separate consent form and will be sent securely to the Department of Human Services who holds this information confidentially. If your child is on two Medicare cards, please note both numbers on the consent form and provide both cardholder's signature. When your child turns 14 years old, they will be required to complete and sign an MBS and PBS (DHS) consent form.

6. **What are my child's alternatives to taking part?**
   Participation in a research project is voluntary. Your child does not have to take part in this project if you do not want them to.

7. **Can my child withdraw from the project?**
   If you give your consent and change your mind, your child can withdraw from the project. You do not need to tell us the reason why you or your child want to stop being in the project. If your child leaves the project we will use any information already collected unless you tell us not to.

8. **What are the possible benefits for my child and other people in the future?**
   If you are in the **Treatment Program Group**, we hope that your child will have less anxiety and this may benefit his/her wellbeing. This may also benefit both your own wellbeing and also your family. If you are in the **Usual Care Group**, you and your child will not receive any direct benefit.

   We are doing this project to see if the **Calm Kids** program works or not. This means that we can't guarantee that your child and family will benefit, even if you are in the **Treatment Program Group.**

9. **What are the possible risks or inconveniences?**
   The survey questions should not cause you any distress. If you feel anxious about any of the questions you don't have to answer them. If you are in the **Treatment Program Group** attending 10 appointments the time commitment may be an inconvenience.

10. How will our information be kept confidential?

In this study we will collect and use personal and health information about your child for research purposes. Any information that we collect will be treated as confidential, and we can only disclose information with your permission except as required by law.

The following people may access information collected as part of this research project:

- The research team involved with this project;
- The Royal Children's Hospital Human Research Ethics Committee

The information will be re-identifiable. This means that we will remove your child's name and give the information a special code number. Only the research team can match your child's name to their code number, if it is necessary to do so.

In accordance with relevant Australian and/or Victorian privacy and other relevant laws, you have the right to access and correct the information we collect and store about you. Please contact us if you would like to access this information.

We are required to keep information collected as part of a research project for a certain length of time. Because the participants in this project are under 18 years old, we must keep information until a participant turns 25 years old. The research information may be destroyed or kept indefinitely in secure storage after this time. The hard copies of the DHS consent forms and data will be kept for seven years from the date of collection. After this time the paper copies will be shredded in a manner that makes reindentification of the data impossible, and the electronic MBS and PBS data will be deleted. Results from this study may be presented at conferences or published in medical journals. When we write or talk about the results of this project, information will be provided in such a way that your child cannot be identified.

11. Will we be informed of the results when Calm Kids finishes?

We will send you a summary of the **Calm Kids** Study results at the end of this project. This summary will give overall results of this project – individual families will not be identified.

12. Who can I contact if I have any questions or concerns?

You can contact the **Calm Kids** study team by phone or email:

Phone: **(03) 9936 6306**

Email: **calm.kids@mcri.edu.au**

Thank you again for taking the time to read this booklet. We hope you and your child will join **Calm Kids**. If so, we hope you both find it a rewarding experience.

Principal Investigator
Dr Emma Sciberras

If you have any concerns and/or complaints about the project, the way it is being conducted or your rights as a research participant, and would like to speak to someone independent of the project, please contact the Director of Research Development & Ethics the Royal Children's Hospital by phone on (03) 9345 5044. Please quote HREC number: 35164

PARENT/GUARDIAN COPY

ID # ☐☐☐☐

PARENT/GUARDIAN CONSENT FORM

HREC Project Number	35164		
Research Project Title	Calm Kids Study		
Version Number	10	Version Date	16/12/2016

- I have read, or had read to me in a language I understand, the information statement version listed above and I understand its contents.
- I believe I understand the purpose, extent and possible risks of my child's involvement in this project.
- I voluntarily consent for my child to take part in this research project.
- I have had an opportunity to ask questions and I am satisfied with the answers I have received.
- I understand that this project has been approved by The Royal Children's Hospital Melbourne Human Research Ethics Committee and will be carried out in line with the National Statement on Ethical Conduct in Human Research (2007).
- I understand I will receive a copy of this Information Statement and Consent Form.

☐ I do<br>☐ I do not	voluntarily consent for me and my child to take part in the Calm Kids research study.

Optional consent

☐ Yes I do	☐ I do not	consent to the researchers contacting my child's school teacher
☐ Yes I do	☐ I do not	consent to re-contact for future research
☐ Yes I do	☐ I do not	consent to storing and using my research information for future use in ethically-approved research projects related to ADHD and other neurodevelopmental disorders
☐ Yes I do	☐ I do not	consent for VCAA (Victorian Curriculum and Assessment Authority) to release my child's years 3, 5,7 NAPLAN results to the Calm Kids study
☐ Yes I do	☐ I do not	consent to Calm Kids accessing my child's healthcare (MBS) and medicines (PBS) data from the Department of Human Services.

____

Child's Name

____

Parent/Guardian Name Parent/Guardian Signature Date

Note: All parties signing the Consent Form must date their own signature.

CALM KIDS COPY

ID # ☐☐☐☐

PARENT/GUARDIAN CONSENT FORM

HREC Project Number	35164		
Research Project Title	Calm Kids Study		
Version Number	10	Version Date	16/12/2016

- I have read, or had read to me in my first language, the information statement version listed above and I understand its contents.
- I believe I understand the purpose, extent and possible risks of my child's involvement in this project.
- I voluntarily consent for my child to take part in this research project.
- I have had an opportunity to ask questions and I am satisfied with the answers I have received.
- I understand that this project has been approved by The Royal Children's Hospital Melbourne Human Research Ethics Committee and will be carried out in line with the National Statement on Ethical Conduct in Human Research (2007).
- I understand I will receive a copy of this Information Statement and Consent Form.

☐ I do<br>☐ I do not	voluntarily consent for me and my child to take part in the Calm Kids research study.

Optional consent

☐ Yes I do	☐ I do not	consent to the researchers contacting my child's school teacher
☐ Yes I do	☐ I do not	consent to re-contact for future research
☐ Yes I do	☐ I do not	consent to storing and using my research information for future use in ethically-approved research projects related to ADHD and other neurodevelopmental disorders
☐ Yes I do	☐ I do not	consent for VCAA (Victorian Curriculum and Assessment Authority) to release my child's years 3, 5,7 NAPLAN results to the Calm Kids study
☐ Yes I do	☐ I do not	consent to Calm Kids accessing my child's healthcare (MBS) and medicines (PBS)data from the Department of Human Services.

____
Child's Name

____
Parent/Guardian Name Parent/Guardian Signature Date

Note: All parties signing the Consent Form must date their own signature.
